# Spectral Photon-Counting Computed Tomography: A Review on Technical Principles and Clinical Applications

**DOI:** 10.3390/jimaging8040112

**Published:** 2022-04-15

**Authors:** Mario Tortora, Laura Gemini, Imma D’Iglio, Lorenzo Ugga, Gaia Spadarella, Renato Cuocolo

**Affiliations:** 1 Department of Advanced Biomedical Sciences, University of Naples “Federico II”, Via Sergio Pansini 5, 80131 Naples, Italy; mario.tortora@ymail.com (M.T.); laura.gemini93@gmail.com (L.G.); diglio.imma@gmail.com (I.D.); lorenzo.ugga@unina.it (L.U.); gaia.spadarella@gmail.com (G.S.); 2 Department of Clinical Medicine and Surgery, University of Naples “Federico II”, Via Sergio Pansini 5, 80131 Naples, Italy; 3 Department of Medicine, Surgery and Dentistry, University of Salerno, Via Salvador Allende 43, 84081 Baronissi, Italy

**Keywords:** photon-counting computed tomography, diagnostic imaging, CT imaging

## Abstract

Photon-counting computed tomography (CT) is a technology that has attracted increasing interest in recent years since, thanks to new-generation detectors, it holds the promise to radically change the clinical use of CT imaging. Photon-counting detectors overcome the major limitations of conventional CT detectors by providing very high spatial resolution without electronic noise, providing a higher contrast-to-noise ratio, and optimizing spectral images. Additionally, photon-counting CT can lead to reduced radiation exposure, reconstruction of higher spatial resolution images, reduction of image artifacts, optimization of the use of contrast agents, and create new opportunities for quantitative imaging. The aim of this review is to briefly explain the technical principles of photon-counting CT and, more extensively, the potential clinical applications of this technology.

## 1. Introduction

The aim of this article is to present one of the most promising new technologies that has been recently introduced in the field of medical imaging, spectral photon-counting computed tomography (SPCCT). This method has been demonstrated to be superior to current generation computed tomography (CT) scanners, based on double-source or dual-energy CT (DECT) technology, and introduced in clinical practice in 2005 [1], whose applications are well known in the literature [2,3,4,5,6]. In order to appreciate the advantages and possible novel clinical applications of SPCCT, it is necessary to be familiar with its basic physical principles and main differences from conventional CT and DECT. An overview of the state of the art of SPCCT technology is now proposed and followed by a discussion of potential clinical applications.

## 2. Technical Principles

**Figure 1:**
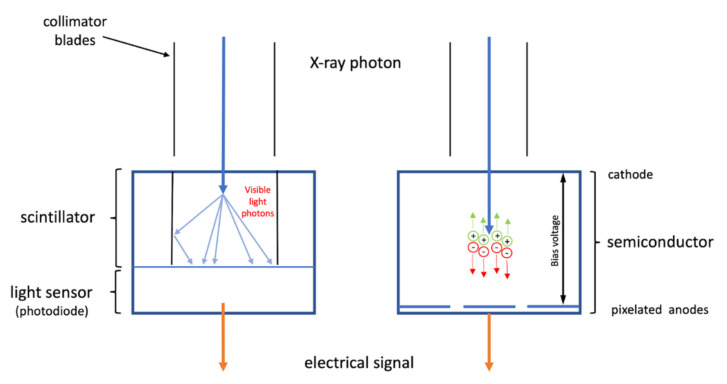
Schematic representation of two different CT systems as follows: on the left, an integrated energy detector (EIDs) with scintillator and septa. The X-rays absorbed by the scintillator are converted into visible light, and a swarm of photons is absorbed by a photodiode that generates an electrical signal. On the right, a photon-counting detector (PCDs) that directly converts X-rays into an electrical signal.

CT devices currently in use in clinical practice employ energy-integrating detectors (EIDs) equipped with scintillator elements and septa. The incident X-rays are absorbed in a scintillator, which converts the X-rays into visible light. This process generates a shower of visible light secondary photons. These are absorbed by a photodiode that generates an electrical signal proportional to the total deposited energy, including electronic noise [7]. Septa are separate detector elements and are crucial to avoid light photon leakage between them; however, they limit the geometric dose efficiency (Figure 1, left panel) [8,9]. Photon-counting detectors (PCDs) convert individual X-ray photons directly into an electrical signal without first transforming them into visible light. The PCDs are made by coupling a semiconductor sensor with a readout circuit. Each photon interacting with the sensor produces electron-hole pairs, which drift towards the electrodes under the effect of a bias voltage applied to the sensor (Figure 1, right panel). The charge carriers collected by the pixels generate an electrical signal with a height proportional to the energy deposited by the interacting photon. By implementing one or more energy thresholds, PCDs can sort the incoming photons according to their energy, excluding the electronic noise. Ideally, an optimal system would be one where the charge carriers produced by each photon are collected by a single pixel. When the charge cloud is produced by a single interaction spread over multiple pixels, there is a reduction in spatial resolution (blurred image) and the contrast-to-noise ratio [10]. This issue, called charge sharing, also affects the energy resolution of a PCD, and it is enhanced as the ratio of sensor thickness to pixel size increases. To limit this effect, CdTe or CZT sensors are preferred to silicon sensors. In fact, if compared to silicon, the high atomic number and density of CdTe and CZT allow for a high detection efficiency with a reduced thickness [11]. Moreover, essential for system optimization is a fast detector able to count single photons [12,13,14]. In fact, pulse pileup may determine a loss of photon count, increasing the image noise. This phenomenon appears as multiple photons rapidly reach the sensor, causing overlapping photon pulses [15]. As stated above, this effect can be minimized by designing smaller pixels and faster counters [16,17,18].

The spatial resolution obtainable with any conventional CT detector is mainly determined by the detector element size, with smaller ones improving the spatial resolution. Since PCDs do not have scintillators and septa, they can be fabricated with smaller elements compared to EIDs [19,20].

Spectral data can be employed through the following two mechanisms: the weighting of energy and the decomposition of the material.

**Figure 2:**
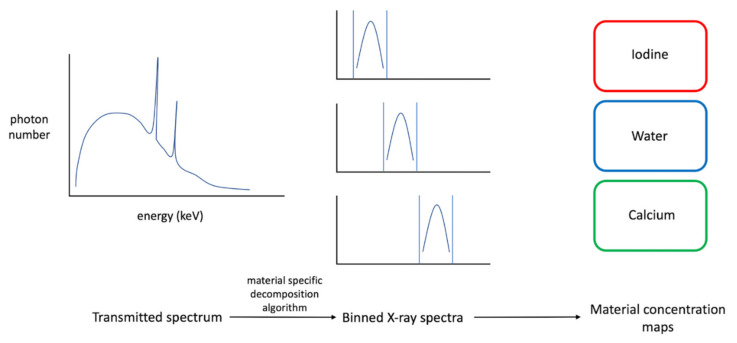
Graphical representation of the ability of the photon-counting system to determine different energy spectra. In particular, the system is capable, by subtracting the raw CT data with defined low-energy thresholds to obtain differentiated data based on the “energy bin” to which it belongs and produce different maps based on the concentration of the materials under examination after spectrum processing by specific material decomposition algorithm (e.g., iodine, water, and calcium).

With the first, greater weights are assigned to specific energy bins in order to improve image quality. As an example, by increasing the weight of low-energy bins, it would be possible to increase the contrast-to-noise ratio (CNR) between soft tissues [21,22]. Several image- and projection-based weighting techniques have been proposed to also increase the visibility of contrast agents and correct for beam-hardening artifacts [23,24,25]. Basis material decomposition algorithms assume that absorption can be modeled as a linear combination of bases [26]. The number of bases that can be selected is limited by the number of spectral data (e.g., with N spectral data, a maximum of N bases can be selected). By imposing mass or volume conservation constraints, the number of bases can be extended to N + 1). This generates a set of base image maps, each of which contains the equivalent material concentration on a voxel-by-voxel basis. These images can be used to directly assess the distribution of a certain element within the image, such as a contrast medium for iodine/water basis (Figure 2). In addition, this technology also allows for virtual images to be generated either in monochrome, without contrast, or with color overlays.

Since full energy-dependent attenuation is considered, the resulting images can, in theory, be completely free of beam-hardening artifacts [27,28,29,30]. Regarding artifacts, SPCCT also has the potential to reduce blooming by means of improved spatial resolution and material decomposition [7]. As we will see later, this is relevant for several applications, such as cardiovascular imaging.

In recent years, numerous prototypes of CT scanners with photon-counting technology have been tested [11,31]. These experiments have confirmed, compared to conventional devices, an improvement in terms of energy weighting and a clear reduction of electronic noise even at low radiation exposure doses. This translates to better imaging resolution paired with a dramatic radiation dose reduction. With reference to the use of contrast media, the SPCCT guarantees the unique opportunity to use multiple contrast media other than iodine [7]. Among the molecules of greatest scientific interest, we have nanoparticles labeled with gold and platinum [32,33,34,35]. In this regard, it is interesting to outline the differences between SPCCT and DECT. Both methods can discriminate between different contrast agents, but with fundamental basic differences. DECT is limited to discerning only two predefined energy spectra, usually high (140 kV) and low (80 kV) [36,37]. Therefore, using this technology, one can break down attenuations into up to two components [38], as only two measurements per voxel are performed, but by including volume (or mass) conservation constraints, it is possible to extend the decomposition up to three components with only two measurements [39,40].

Using SPCCT, on the other hand, it is possible to discern more than two different contrast agents in each voxel at the time of acquisition, with different pharmacokinetics in the same biological system. This allows for the generation of separate quantitative maps for each component, with up to five photon-counting measurements (six bins) per voxel [41]. The components commonly identified in pre-clinical studies were the following: two non-K-edge materials (e.g., water and iodine) and one or two K-edge materials (e.g., gadolinium and gold) [42,43]. As reported by Si-Mohamed et al. [44], SPCCT is able to accurately decompose the material for both unmixed (iodine, gadolinium, and gold) and mixed (iodine-gadolinium and gadolinium-gold mixtures) solutions. However, there is an underestimation of the contrast power for mixed solutions, and, among the K-edge materials, gadolinium has a greater contrast than gold in relation to the better equilibrium of photons in a range of 120 kV, which determines a more accurate decomposition of this material. Therefore, SPCCT enables multicolor quantitative imaging. As a result, it should be possible to perform imaging of multiple uptake phases of a given tissue/organ within a single slice scan by injecting contrast agents sequentially at different time points.

## 3. Clinical Applications

### 3.1. Head and Neck Imaging

SPCCT has the potential to improve the quality of carotid and intracranial angiography imaging compared to conventional single-energy TC and DECT [27]. In particular, it has been reported that this technique minimizes electronic noise and beam-hardening artifacts in internal carotid artery segments close to surrounding bone [45]. Spectral material decomposition is feasible for vascular imaging in this human district [36], with the technical advantages mentioned above and a reduction in overall radiation dose [20]. Another field of application of SPCCT in the head and neck district is the staging of laryngeal and hypo-pharyngeal cancer, for which it is essential to identify the degree of invasion of the laryngeal cartilages. Since the density of non-ossified laryngeal cartilage is similar to that of tumors in conventional CT imaging, greater spatial resolution, as well as material decomposition and differentiation techniques allowed by SPCCT, may lead to improved lesion local staging [46].

### 3.2. Temporal Bone Imaging

A high spatial resolution, guaranteed by SPCCT, is essential for temporal bone imaging, where a high-resolution study of small structures such as auditory ossicles is required. Initial studies were performed in pigs’ cadavers [47], followed by phantom and cadaver studies [48,49]. The results showed that SPCCT guarantees the clear visualization of crucial anatomical structures, such as the stapes superstructure, with a lower radiation dose than conventional CT scanners.

### 3.3. Chest Imaging

Preclinical studies have found that SPCCT has the potential to improve the detection of pulmonary nodules and the assessment of other lung structures [50]. In particular, the high spatial resolution allowed by SPCCT, compared to conventional CT scanners in current clinical use, leads to a more precise assessment of nodules and the smallest pulmonary structures such as the terminal divisions of the bronchial tree and the interstitium. Altogether, SPCCT guarantees a strong improvement in detectability of both lesions with low-contrast compared to surrounding parenchyma (e.g., ground glass nodule) and lesions with high-contrast (e.g., solid nodule). For example, SPCCT has proven superior to DECT for the detection of solitary pulmonary nodules, and it is worth noting that a less than 2 mm-diameter ground glass nodule surrounded by an extension ring was clearly seen only with SPCCT, confirming the potential information gain with this technology [51]. Compared to DECT [52], the lower electronic noise level and higher spatial resolution of SPCCT are confirmed in this domain. Several studies [51,53] support the technical improvements of PCDs, making SPCCT a promising tool for radiation dose optimization, which is a crucial aspect in improving the risk-benefit ratio of CT lung cancer screening. For the study of pulmonary parenchyma, SPCCT can revolutionize the role of imaging in the detection of interstitial lung disease and pathological key signs including intra-lobular reticulations, bronchiectasis, and honeycombing [54,55,56]. Distal airways and bronchial imaging still represent challenges for even the most experienced radiologists. Recent studies [50,57] have shown an improvement in the detection of high-order bronchi compared with conventional CT systems. In addition, bronchi, bronchioles, vessels, and walls of bronchioles could be visualized more distinctly with SPCCT compared to high-resolution CT. Last but not least, in reference to pulmonary vascular diseases in the setting of the coronavirus disease 19 pandemic, there is hope for better monitoring of the distal pulmonary vascular involvement [58].

### 3.4. Breast Imaging

Currently, SPCCT is not used for breast imaging, although numerous experimental studies have investigated how PCDs in a dedicated breast CT system could guarantee advantages for the detection of small lesions and the differentiation of soft tissues [59,60]. The use of PCDs in the detection of breast cancer bone metastases is included in the following section on musculoskeletal imaging.

### 3.5. Cardiovascular Imaging

Conventional CT systems can rule out coronary artery disease in relatively large normal vessels, but their specificity remains low, as precisely measuring the degree of stenosis requires very high spatial resolution. Additionally, calcified plaques are often affected by blooming artifacts. The increased spatial resolution of SPCCT allows for increased detectability and more accurate coronary artery calcium (CAC) volume estimation with the classic Agatston score and the minimization of blooming artifacts. These conclusions are the result of observations in phantom studies as well as in-vivo human experiences [61,62].

Another interesting application of this technology is to determine the extent of damage in a myocardial infarction using a double-contrast agent. Cardiac imaging with a multi-contrast agent is possible by injecting the gadolinium-based contrast 10 min before the SPCCT and the iodinated contrast immediately before the scan. Gadolinium and iodine maps are then obtained using the previously mentioned material decomposition technique. For myocardial infarct versus remote myocardium, the contrast-to-noise ratio was maximized on the gadolinium-enhanced maps, while for myocardial infarct versus the blood pool, it was optimal on iodine maps. Combined first-pass iodine and late gadolinium maps enabled the quantitative separation of the blood pool, scar, and remote myocardium with accurate scar delineation [63]. Remaining in the domain of coronary heart disease, the use of these techniques is also very promising for the study of endovascular stents. There are specific technical issues when imaging coronary stents with current CT scanners in clinical practice. These include metallic, blooming, and beam-hardening artifacts, in addition to partial volume effects due to relatively low spatial resolution. With conventional CT scanners, the lumen of the coronary stent can be visualized accurately in a still unsatisfactory percentage of cases [64,65,66,67]. In the study by Mannil et al. [68], SPCCT was directly compared with the best available detection technology currently available in the clinical setting. Thus, the following best results were observed for PCDs-based CT technology with significantly improved image quality: 16% better lumen display within the stent; fewer blooming and partial volume artifacts that are reflected in a 37% lower increase in the attenuation of the lumen inside the stent. In the assessment of stent features, SPCCT has offered superior image quality, which can result in new diagnostic capabilities and potentially enable novel applications. A clear assessment of the stenosis internal to the stent and the adjacent residual lumen was possible, which allowed highly reliable non-invasive assessments of the actual grade and extent of the stenosis [69]. Although drug-eluting stents significantly reduce the number of in-stent stenoses [70], these are still highly relevant complications [71], negatively impacting clinical outcomes [72]. Future screening with SPCCT could be an option to identify these complications and select patients in need of repeat angioplasty.

Another application in the vascular field concerns the determination of aortic stent or graft endoleaks. In this setting, SPCCT in combination with a dual-contrast agent injection protocol can reduce the conventional TC acquisition phases without sacrificing diagnostic accuracy for endoleak detection and classification. It is thus possible to capture the fluid dynamics of the endoleaks and allow a confident differential diagnosis with intra-aneurysmal calcifications in a single CT scan. This new approach will realize a relevant reduction in the radiation burden for patients undergoing CT surveillance after aortic endovascular aneurysm repair [73].

### 3.6. Abdominal Imaging

Diagnostic imaging of liver disease is very common in clinical practice, often requiring a dedicated multiphase CT acquisition protocol for both benign and malignant lesions, such as hemangiomas, hepatocellular carcinoma (HCC), and metastases [74,75,76]. A retrospective in-silico study assessed SPCCT’s ability to depict characteristic arterial and portal venous enhancement with a single scan, allowing for streamlined lesion detection and characterization, again with additional benefits in terms of reduced radiation exposure dose [77]. As previously stated, this is due to multi-color imaging allowed by SPCCT, producing multiple image maps containing different information [78]. Regarding the liver, preclinical studies on rabbits focused on the administration of iodine- and gadolinium-based contrast mediums at different time points prior to CT scanning, leading the first to provide information usually obtained in the portal phase while the second to arterial phase images.

Peritoneal metastases are indicative of terminal disease in most abdominal malignancies. Still, over the past decades, the development of cytoreductive surgery with or without intraperitoneal chemotherapy has led to improved patient survival for this population [79,80,81]. For example, this approach has been tested in colorectal cancer, obtaining improved oncological outcomes [82]. In this context, preoperative assessment of the peritoneal tumor burden is essential for the selection of patients eligible for potentially curative treatment [83,84]. Magnetic resonance imaging (MRI) and hybrid imaging pairing positron emission tomography and CT have shown good diagnostic performance, but these modalities are mainly used as a complement to CT evaluation [83,85,86,87]. However, even though CT imaging offers a high spatial and temporal resolution, its main drawback is the lack of contrast resolution [88,89]. Therefore, current peritoneal imaging could also take advantage of the promising opportunities provided by SPCCT. An initial experimental study in rats [90], using a double-contrast agent injection protocol (i.e., intravenous iodine and intraperitoneal gadolinium or the reverse protocol) into the peritoneal and vascular compartments at different administration times, demonstrated a much greater sensitivity (65%) of SPCCT for small lesions (defined as <5 mm) compared to a sensitivity of 11% reported by Koh et al. using conventional CT [88]. If these results can be translated to clinical practice, the higher sensitivity provided by SPCCT could avoid unnecessary “open-close” surgical procedures.

### 3.7. Musculoskeletal Imaging

Articular cartilage is a highly specialized tissue, composed of water, collagen fibers, and proteoglycans [91,92]. Due to its avascular nature and limited capability for regeneration, an acute joint injury may induce post-traumatic osteoarthritis with a loss of proteoglycans, breakdown of fibers, and an increase in water content [93,94]. By estimating the concentration of two contrast agents (non-ionic gadolinium-based gadoteridol and cationic iodinated CA4+) [36], SPCCT could provide information directly indicative of the water and proteoglycan content in the articular cartilage [95]. Therefore, contrast-enhanced SPCCT imaging has potential use for the assessment of articular cartilage health and monitoring the evolution of osteoarthritis [96].

Osteoarthritis represents the most common cause of disability, and it can be managed by arthroplasty, especially for the knee [97]. Despite its frequent use, almost 20% of patients report residual symptoms and functional limitations after surgical treatment [98]. Establishing the cause of symptoms and pain is fundamental to proper management, as revision surgery with an uncertain diagnosis has a poor outcome [99]. The currently available imaging techniques are important, but each modality presents its own limitations. For example, radiography and fluoroscopy cannot delineate soft tissues; ultrasound may depict effusions and soft tissue structures but cannot satisfactorily evaluate the prosthesis or surrounding bony structure; conventional CT is limited by beam-hardening artifacts; MRI is subject to metallic susceptibility artifacts caused by the prosthesis [100]. In a study reported by Lau et al. [101], SPCCT could clearly identify polyethylene inserts and metallic tray wear as the causes of prosthesis failure. These could not be identified on standard pre-operative imaging.

SPCCT technology has other potential applications in the domain of musculoskeletal imaging. For example, its material decomposition capabilities can be used to reconstruct calcium-free images (i.e., non-calcium virtual images), allowing the evaluation of bone edema without the need for MRI [102]. Furthermore, SPCCT can detect and characterize the deposition of calcium crystals within the articular cartilage with high sensitivity and relatively good specificity, as reported in the recent literature [103]. SPCCT is also the only imaging technology capable of distinguishing between calcium pyrophosphate and hydroxyapatite deposits, both when present in isolation and coexisting in the same joint cartilage. This could provide new insights into the pathogenesis of crystal deposition rheumatic diseases and aid in identifying new potential therapeutic targets.

Regarding the field of oncology, bone metastases are very frequent. Taking breast cancer as an example, bone involvement is the common distant form of metastasis [104,105]. These lesions cause high morbidity with mortality hazard ratios of 5–6 compared to patients without metastatic bone lesions [106]. In this regard, it is essential to correctly diagnose their presence to guide the most correct therapeutic process. SPCCT may allow us to identify smaller bone lesions with submillimeter spatial resolution, potentially leading to a more precocious diagnosis. Its properties could also be used to differentiate between true tumor growth and changes associated with therapy, mainly in a sclerotic sense (i.e., pseudo-progression). In particular, SPCCT reaches resolutions comparable with bone trabecular diameters [107] and the resulting high-resolution images may allow for the detection of metastatic changes in shorter intervals compared to conventional CT and MRI imaging [108].

## 4. Conclusions

SPCCT has the potential to dramatically alter the clinical use of CT in the coming years. By using energy-resolving detectors instead of EID, these new CT systems can substantially reduce image noise, increase the spatial resolution, and use K-edge imaging to measure the concentration of specific elements with reduced radiation doses compared to current standards. These properties can be useful in the evaluation of multiple anatomical districts, where it is necessary to identify small physiological and pathological structures, as well as for the use of different contrast agents, even concurrently, as shown in our review.

## Data Availability

Data sharing not applicable.
